# Motivation Research on the Content Creation Behaviour of Young Adults in Anxiety Disorder Online Communities

**DOI:** 10.3390/ijerph18179187

**Published:** 2021-08-31

**Authors:** Jingfang Liu, Yafei Liu

**Affiliations:** School of Management, Shanghai University, 99 Shangda Road, Shanghai 200444, China; jingfangliu@shu.edu.cn

**Keywords:** young adults, anxiety disorder, online mental health community, content creation behaviour, behavioural motivation, empathy

## Abstract

With the advancements in science and technology and the improvement of medical care, mental health problems are receiving increasing attention. Increasing numbers of children, adolescents, and young adults are susceptible to anxiety. This paper assesses young adults based on self-determination theory and the theory of planned behaviour to determine the intrinsic and extrinsic motivations and mediating variables behind young adults’ content creation behaviour within anxiety disorder online communities (ADOCs). In addition, the paper introduces empathy as a moderating variable, builds a model of the content creation behavioural motivation of young adults, studies the motivation behind young adults’ content creation behaviour in ADOCs, and determines the moderating effect of empathy on young adults’ content creation behaviour. The research data were obtained using a questionnaire survey, and the SmartPLS structural equation model was used for empirical analysis. The study found that expressing one’s anxiety was the most obvious motivation, the content creation intention of young adults significantly positively affected their content creation behaviour, perceived enjoyment motivation had a significant negative influence on young adults’ intention to create content, reward motivation had no significant influence on the content creation intention of young adults, other motivations had significant positive influences on young adults’ content creation intention, and empathy only had a significant negative moderating effect on the relationship between self-efficacy and young adults’ content creation intention. This study not only enriches and expands research on motivation theory but also has practical significance for the improvement and active development of ADOCs.

## 1. Introduction

In recent years, due to various social work and life pressures, an increasing number of individuals have been found to suffer from mental illnesses, such as schizophrenia, depression, anxiety disorders, obsessive-compulsive disorder, and others; mental illnesses tend to affect younger people [1]. Many young adults suffer from anxiety disorders [2], especially in large cities, because their stress-bearing abilities are limited. Once the level of anxiety exceeds one’s ability to cope, self-harm and suicide attempts may occur [3]. Awareness of the seriousness of mental illness is increasing, as well as the awareness of the importance of timely diagnosis and treatment. 

However, patients with mental illnesses such as anxiety are often unwilling to participate in offline doctor–patient communication due to the stigma associated with their mental illness [4]. With the development of e-health, online health communities provide users with convenient channels for seeking health information and online diagnosis and treatment [5]. In recent years, a variety of online health communities have emerged at home and abroad, such as WebMD, MedHelp, and Yahoo! Health in foreign countries and 39.net, haodf.com, chunyuyisheng.com, and dxy.cn in China. Anxiety patients can obtain health information, share their treatment experiences, and seek emotional support through online health communities [5,6]. Posting and commenting are the main avenues by which users participate in online communities, and they are also key to maintaining the success of such communities. The proportion of anxiety patients among young people is gradually increasing [2]. Young adults have become the main demographic responsible for content creation in online health communities [7]. However, the factors motivating young adults to post content in anxiety disorder online communities (ADOCs) remain unknown. It is necessary to study the motivation behind young adults’ content creation behaviour in ADOCs. Therefore, this paper aims to reveal the main reasons for which young adults create anxiety content within online communities. For this purpose, we study the motivation behind young adult users’ content creation behaviours in ADOCs. 

## 2. Literature Review

According to the results of the “2019 World Health Statistics Report” by the WHO, the number of people who died from chronic non-communicable diseases in 2016 accounted for 71% of total deaths [8]. Chronic non-communicable diseases account for a high proportion of deaths each year, causing a serious social burden. The prevention, control, and management of chronic non-communicable diseases have been hot topics in research and the focus of domestic and foreign scholars in recent years. Research on chronic diseases mainly focuses on chronic physiological diseases, such as diabetes [9], hypertension [10], and breast cancer [11,12,13]. For example, Zhang S et al. studied the personal health of members of the online cancer community [14]. Some scholars have used breast cancer simulation models to study the role of intervention and evaluate the incidence, survival rate, and mortality associated with breast cancer after intervention [12,13]. In addition, Tao Zheng et al. used a variety of machine learning algorithms and frameworks to identify whether subjects had diabetes [9]. There are relatively few studies on chronic mental illness at home and abroad. At present, the most common research related to chronic mental illness is the study of depression [15]. 

The online health community provides an effective channel for patients with chronic diseases to obtain health information and discuss disease-related topics [5]. The emergence of a series of online health communities has attracted a large number of scholars at home and abroad to conduct research on online health communities. The participation process of internet users in online communities is often accompanied by posts, comments, replies, and other content creation behaviours or content consumption behaviours, such as browsing and searching. Content consumption behaviours, such as browsing and searching, are not easy to study. Replies and comments can leave traces in an online community. This is a good source of data and information for scholars to conduct academic research. Research on user behaviour in online communities mainly includes help-seeking behaviour [16], knowledge sharing behaviour [17,18], information adoption behaviour [19], and continuous use behaviour [20,21]. Golnar Aref-Adib et al. found that most patients with a mental illness seek online mental health information, such as disease diagnosis and medication [16]. JiahuaJin et al. studied the influences of information quality, emotional support, and source credibility on patients’ medical information adoption behaviour based on dual-process theory and an information adoption model [19]. Ma et al. studied information on Facebook and other social media to explore the intrinsic motivation behind users’ knowledge sharing behaviour. The research found that network attachment motivation and network relationship commitment have significant influences on knowledge sharing and that altruism has a direct and significant effect on knowledge sharing [18]. Jeroen Stragier et al. studied the reasons for the continuous use of online fitness communities using a questionnaire survey of new users and old users, and the results showed that self-regulation is the main factor influencing new users’ continuous use of an online fitness community, while old users are motivated by interaction and enjoyment [21]. 

Overall, through this literature review, this study finds that domestic and foreign research on online health communities mostly focuses on chronic physical diseases, including diabetes, high blood pressure, breast cancer, obesity, etc. Studies on online mental health communities are relatively few. In particular, few scholars study users in ADOCs. Although increasing numbers of young adults are becoming susceptible to anxiety [1], few scholars study young adults [22]. Therefore, this paper focuses on a group of young adult users in ADOCs. In addition, most of the literature only studies one kind of content creation behaviour, such as knowledge sharing behaviour, user reply behaviour, help-seeking behaviour, etc., which has certain shortcomings. Meanwhile, few studies have investigated user behavioural motivations from the perspective of moderating effects. Therefore, to explore the main reasons that motivate young adults to create content in online communities for anxiety disorders, we study the motivation behind young adult users’ content creation behaviours in ADOCs. This paper takes young adults of ADOCs as the research object, and we use self-determination theory and planned behaviour theory to construct a model of the content creation behaviour of young adults with anxiety. In addition, this paper also analyses the moderating effects on young adult users’ content creation behaviour from the perspective of empathy. 

## 3. Theoretical Background and Research Hypotheses

### 3.1. Theoretical Background

The American psychologists Deci and Ryan proposed self-determination theory (SDT), which is a kind of cognitive motivation theory that has been widely applied in recent years, on the basis of traditional cognitive evaluation theory. The theory divides individual behavioural motivations into extrinsic motivations and intrinsic motivations [23]. Intrinsic motivation means that the reason that an individual performs an activity is to meet their own needs or to obtain enjoyment from it, which is autonomous or self-determined [23,24]. On the contrary, extrinsic motivation means that an individual performs an activity due to external reasons in order to attain an independent outcome [23,25]. Self-determination theory emphasises the intrinsic motivations of individuals more and assumes that an individual has three basic psychological needs: autonomy, competence, and relatedness. Autonomy indicates that the individual can freely choose and perform a task or activity according to his or her self-consciousness [26], competence reflects the individual’s inner desire to be able to complete a certain activity, and relatedness reflects the individual’s inner needs for interpersonal relationships [24]. When the three basic psychological needs are met, the individual will be more positive and proactive in certain activities and behaviours. This study proposes that the influencing factors behind users’ content creation behaviour include internal and external factors. Therefore, this study uses self-determination theory to analyse the intrinsic motivations and extrinsic motivations underlying the content creation behaviour of young adults in ADOCs. 

### 3.2. Mediating Variable

The content creation behavioural motivation of young adult users in ADOCs studied in this article belongs to the field of behavioural science, and the theory of planned behaviour (TPB) proposed by Ajzen is one of the most basic and classical theories with which to study individual behaviours and intentions [27]. The theory holds that behavioural intention is a necessary process for any behaviour [27]. Therefore, based on the theory of planned behaviour, this study takes young adult users’ content creation intention as the mediating variable. In this study, young adult users’ content creation intention is defined as young adult users’ intention to create content through posts, comments, and consultations in ADOCs; we analyse the influence of the content creation intention of young adult users in ADOCs on their content creation behaviour. Based on the above, this paper proposes the following hypothesis:

**Hypothesis** **1 (H1).** *Young adults’ content creation intention is positively associated with their content creation behaviour in ADOCs.*

### 3.3. Intrinsic Motivation

Self-determination theory emphasises the intrinsic motivation behind individual behaviour. Intrinsic motivation is based on an individual’s enjoyment or satisfaction [25]. Therefore, this study selects anxiety expression and perceived enjoyment as two of the intrinsic motivations. In addition, based on the three basic psychological needs of individual autonomy, competence, and relationships in self-determination theory, this study selects online communities’ sense of belonging, altruism, and self-efficacy as other intrinsic motivations. Self-determination theory believes that emotional satisfaction is a significant internal factor for self-motivation [26]. Anxiety expression and perceived enjoyment are both processes of self-emotional satisfaction [28,29,30]. Liu’s research found that users vent negative emotions in online health communities, such as anxiety, pain, sadness, etc. Venting can also be understood as a form of catharsis. Generally, venting involves negative emotions, and anxiety is a negative emotion. To a certain extent, proper venting can ease tension, fear, and anxiety; ADOCs provide effective platforms and methods for young adults to vent negative emotions caused by anxiety. This study defines anxiety venting as young adults releasing their anxiety and relieving their psychological pressure in ADOCs. Stragier’s research found that enjoyment motivation is an important factor behind users’ to continued participation in online fitness communities [21]. Perceived enjoyment, as an entertainment motivation, aims at relaxation, leisure, etc. Hsu’s research found that microblog users create and share content for entertainment [31]. However, through interviews with young adult users in the community, it was found that the content posted by them in ADOCs is mostly associated with negative emotions. Therefore, this study proposes that young adult users in ADOCs are more likely to demonstrate the intention to create content such as posting comments when their perceived enjoyment is low. In this paper, perceived enjoyment is defined as young adult users relaxing and obtaining enjoyment by publishing content in ADOCs. According to self-determination theory, the satisfaction of an individual’s internal goals is closely related to their sense of belonging and community [32]. Online communities’ sense of belonging refers to the degree to which users perceive themselves as belonging to the community [33], and the sense of belonging of members in an online community has a certain role in promoting their participation in community activities and contributing knowledge [34]. In this study, online communities’ sense of belonging is defined as the degree to which young adult users in ADOCs perceive that they belong to the community. Altruism is an intrinsic motivation [17]. Individuals are willing to help and enjoy helping others, which reflects their sense of social responsibility and mission [35] and also reflects their need for autonomy. Studies have found that individuals who display altruism are more willing to contribute knowledge within virtual communities [36]. This paper defines altruism as the degree to which young adult users in ADOCs are willing to help and enjoy helping others to solve problems. Self-determination theory emphasises personal self-motivation [26]. Individuals who display high levels of self-efficacy have more powerful self-motivation capabilities [37]. Self-efficacy refers to the degree of confidence with which an individual can provide useful information to others [38], which also reflects the individual’s need for competency. Related research shows that individuals who display high levels of self-efficacy are more likely to respond to and help others [37]. In this paper, self-efficacy is defined as the degree of confidence with which young adult users in ADOCs can provide useful information to others. Based on the above, this paper proposes the following hypotheses:

**Hypothesis** **2 (H2).** *Anxiety expression is positively associated with young adults’ content creation intention in ADOCs.*

**Hypothesis** **3 (H3).** *Perceived enjoyment is negatively associated with young adults’ content creation intention in ADOCs.*

**Hypothesis** **4 (H4).** *Online communities’ sense of belonging is positively associated with young adults’ content creation intention in ADOCs.*

**Hypothesis** **5 (H5).** *Altruism is positively associated with young adults’ content creation intention in ADOCs.*

**Hypothesis** **6 (H6).** *Self-efficacy is positively associated with young adults’ content creation intention in ADOCs.*

### 3.4. Extrinsic Motivation

Self-determination theory believes that individuals may perform certain activities due to external factors [39], such as money, scores, reputation, knowledge, and social interaction. There is a great deal of content in the online health community about seeking health-related information [40]. Obtaining anxiety information reflects the individual’s demand for external health knowledge. Chiu’s research found that access to health information is a significant reason that people post in online health communities [40]. In this paper, anxiety information acquisition is defined as young adults in ADOCs seeking, acquiring, and learning about mental health. Reciprocity reflects the external relationship where in both sides benefit from each other and the two sides of interaction are mutual and fair [41]. The research of Wasko et al. showed that reciprocity is a significant factor involved in user interaction and knowledge contribution in virtual communities [42]. Therefore, this study takes reciprocity motivation as one of the external motivations. Reciprocity motivation is defined as the expectation of young adult users in ADOCs to receive responses or rewards while communicating and providing mental health knowledge. Self-determination theory believes that reward motivation is the satisfaction of external goals [30]. Yoon et al. found that by giving a certain amount of money, other material, or honour rewards, people’s enthusiasm for certain activities and tasks is increased [43]. In this paper, reward motivation is defined as the expectation of receiving points, honour, money, or other rewards among young adult users in ADOCs when they create content. ADOCs provide young adults with channels and opportunities to communicate with others who experience the same symptoms as them while also expanding their interpersonal relationships. Related research shows that users have certain social motivations to participate in the community, and users with similar experiences or the same health conditions are more likely to form social relationships [44]. Kim et al. also found that expectations of social interaction are a significant factor involved in individuals’ participation in online communities [45]. In this paper, social motivation is defined as young adult users in ADOCs communicating and interacting with other members to form social relationships with them. Based on the above, this paper proposes the following hypotheses:

**Hypothesis** **7 (H7).** *Anxiety information acquisition is positively associated with young adults’ content creation intention in ADOCs.*

**Hypothesis** **8 (H8).** *Reciprocity motivation is positively associated with young adults’ content creation intention in ADOCs.*

**Hypothesis** **9 (H9).** *Reward motivation is positively associated with young adults’ content creation intention in ADOCs.*

**Hypothesis** **10 (H10).** *Social motivation is positively associated with young adults’ content creation intention in ADOCs.*

### 3.5. Moderating Variable—Empathy

Empathy is a psychological concept that can be understood in many different ways. At present, there is no clear and unified view on the concept of empathy. Some scholars believe that empathy is the feeling of concern for people in need and empathy for another party’s situation [46]. Some scholars also believe that empathy is the ability to correctly perceive others’ feelings and form appropriate empathic responses [47]. The ways in which individuals in online communities respond to others mainly involve content creation behaviours, such as comments, replies, etc. Empathy plays a significant role in social interaction [48]. A large number of studies have shown that empathy can stimulate individual pro-social behaviours [48,49,50], such as sharing knowledge, emotional support, etc. On the one hand, empathy can cause individuals to realise that others need help [51]. Users with high empathy levels are more likely to display an intention to help others and implement pro-social behaviours [52]. On the other hand, individuals with different empathy levels also have different tendencies to obtain social support. Users with high empathy levels are more likely to experience and receive social support [53]. According to previous research, empathy has a certain connection with content creation intention [48], such as replies and comments. However, the role of empathy in the relationship between motivation and the intention behind individual content creation requires further exploration. This study defines empathy as an individual’s emotional perception of the content posted by other users in ADOCs. We introduce empathy as a moderating variable to analyse the relationship among young adults’ empathy, content creation motivations, and intentions. Based on the above, this paper proposes the following hypotheses:

**Hypothesis** **11-1 (H11-1).** *Empathy positively moderates the relationship between anxiety venting and young adults’ content creation intention in ADOCs.*

**Hypothesis** **11-2 (H11-2).** *Empathy positively moderates the relationship between perceived enjoyment and young adults’ content creation intention in ADOCs.*

**Hypothesis** **11-3 (H11-3).** *Empathy positively moderates the relationship between online communities’ sense of belonging and young adults’ content creation intention in ADOCs.*

**Hypothesis** **11-4 (H11-4).** *Empathy positively moderates the relationship between altruism and young adults’ content creation intention in ADOCs.*

**Hypothesis** **11-5 (H11-5).** *Empathy negatively moderates the relationship between self-efficacy and young adults’ content creation intention in ADOCs.*

**Hypothesis** **11-6 (H11-6).** *Empathy positively moderates the relationship between anxiety information acquisition and young adults’ content creation intention in ADOCs.*

**Hypothesis** **11-7 (H11-7).** *Empathy positively moderates the relationship between reciprocity motivation and young adults’ content creation intention in ADOCs.*

**Hypothesis** **11-8 (H11-8).** *Empathy positively moderates the relationship between reward motivation and young adults’ content creation intention in ADOCs.*

**Hypothesis** **11-9 (H11-9).** *Empathy positively moderates the relationship between social motivation and young adults’ content creation intention in ADOCs.*

**Figure 1:**
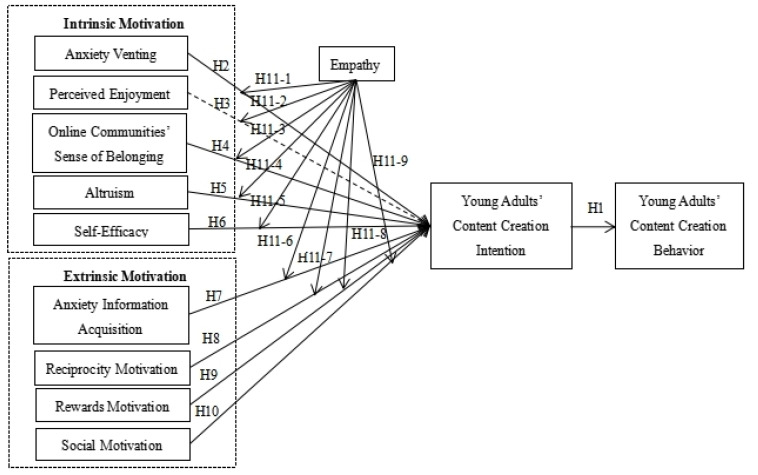
Research model.

In summary, based on self-determination theory, this article divides the behavioural motivations of young adults in ADOCs into internal motivations and external motivations and also introduces empathy as a moderating variable. The constructed model of young adults’ behavioural motivations regarding ADOCs is shown in Figure 1. 

## 4. Materials and Methods

### 4.1. Scale Design

This study uses the questionnaire survey method to conduct an empirical analysis of the proposed hypotheses and research model. The questionnaire contains three parts: screening items, demographic variables, and latent variables. The screening items include use experience, frequency of use, and participation behaviour in ADOCs. The demographic variables include gender, age, education, and living area. The latent variables include anxiety venting (AV), perceived enjoyment (PE), online communities’ sense of belonging (OCSB), altruism (AL), self-efficacy (SE), anxiety information acquisition (AIA), reciprocity motivation (RE), reward motivation (RM), social motivation (SM), empathy (EM), young adults’ content creation intention (YACCI), and young adults’ content creation behaviour (YACCB). All the questionnaire items for the latent variables come from relevant domestic and foreign literature. According to the research object, we adapted the expression in the questionnaire items. The questionnaire in this paper uses a 7-level Likert scale, ranging from very disapproving to very approving. The relevant latent variables and questionnaire items are shown in Table 1. 

To ensure the content validity of the questionnaire, this study conducted a small-scale pre-survey before the questionnaire was officially released. Eight experts in online health-related research fields were selected to fill out and evaluate the questionnaire. They assessed the rationality and necessity of the questionnaire items and confirmed that all the latent variable items were necessary. In addition, based on expert opinions, we appropriately deleted the screening items of the questionnaire and demographic-related items. We also revised the wording of all items in the questionnaire. Finally, 47 items were obtained. 

### 4.2. Data Collection

In this study, the questionnaire was produced and distributed through the Questionnaire Star platform. Due to the recent epidemic, the authors distributed the questionnaire through the internet and selected two ADOCs, the microblog anxiety disorder chat and the Baidu anxiety disorder Tieba, as the research communities for this paper. We briefed the participants of our study at the beginning of the questionnaire and obtained their agreement. The users in the community were paid to fill out the questionnaire, and snowball sampling was used to collect as much questionnaire data as possible. The questionnaire set up screening items such as whether the user had used ADOCs and their use frequency. We used these data to determine whether the participants were ADOC users. 

The collection period was from 29 April 2020 to 18 May 2020. We collected more than 600 questionnaires. To ensure the authenticity and validity of the collected data, based on the screening items of the questionnaire, we eliminated the questionnaires completed by individuals who were not users of ADOCs. In addition, we also excluded questionnaires with too little answer time, missing answers, and logical contradictions. We screened the respondents according to their age, keeping only those under 30 years old. After screening, a total of 334 valid questionnaires were collected. The basic information of the sample is shown in Table 2. 

## 5. Results

In this study, the SmartPLS3.0 software was used for data analysis and validation of the proposed hypotheses and the constructed model. On the basis of ideal reliability and validity, the structural equation model was tested by the partial least squares (PLS) method. In general, the PLS method is suitable for small- and medium-sized sample studies and is more suitable for the analysis of complex models [63]. Since the sample data in this study were small and there were many variables, the model was relatively complex, so the PLS algorithm was suitable for this study [63]. 

### 5.1. Reliability and Validity Test

The reliability and validity of the model needed to be tested before structural model testing, which generally includes reliability analysis and validity analysis. Reliability is used to measure the consistency and stability of the measurement results of a scale. Generally, when Cronbach’s alpha and the composite reliability (CR) reach 0.7, this indicates that the result is acceptable and it meets the reliability requirements [64]. As shown in Table 3, the CRs were all above 0.7, and the Cronbach’s alpha of each variable ranged from 0.726 to 0.957, indicating that the model had good internal consistency. 

Validity, including content validity and structural validity, is used to reflect the validity of the measurement results of a scale. Because all the latent variable items in the questionnaire of this study were based on domestic and foreign literature and because experts in the field of online mental health communities were invited to evaluate the rationality and the wording of the questionnaire, the questionnaire had good content validity. Construct validity includes convergent validity and discriminative validity. In this study, when conducting confirmatory factor analysis (CFA), the model’s convergent validity and discriminative validity were analysed, respectively. Table 3 shows that all factor loadings were greater than 0.7, and the AVE was greater than 0.5, indicating the convergent validity of the model [64,65]. Discriminant validity reflects the correlation coefficient between each latent variable and the other latent variables. As shown in Table 4, the square root of the AVE of each latent variable was greater than the correlation coefficient with the other latent variables, indicating that the model in this study had good discriminant validity [64,65]. 

### 5.2. Structural Model Inspection

#### 5.2.1. Main Effect Test

In this paper, the main effect and the moderating effect were each analysed when the structural model was tested. First, the main effects were tested to analyse the effects of each latent variable. The SmartPLS3.0 software was used to test the hypothesis of the model. The bootstrapping repeated sampling method was used, and the number of sampling times was 5000. The PLS algorithm was used to obtain the value of the model fitting index R^2^ [63]. The results show that the R^2^ of the endogenous latent variable young adults’ content creation intention (YACCI) was 0.726, and the R^2^ of young adults’ content creation behaviour (YACCB) is 0.274. Therefore, the model hada good fitting effect [63]. 

Table 5 lists the path coefficients of each hypothesis and their significance levels. Table 5 shows that the t-statistics of hypotheses H2, H3, H4, H5, H6, H7, H8, H10, and H1 were all greater than 1.96 and the *p*-values were less than 0.05. Therefore, anxiety venting, perceived enjoyment, online communities’ sense of belonging, altruism, self-efficacy, anxiety information acquisition, reciprocal motivation, and social motivation were all significantly related to young adults’ content creation intention in ADOCs. Young adults’ content creation intention had a significant impact on young adults’ content creation behaviour in ADOCs. It can be seen that anxiety venting (β = 0.249, *p* < 0.01) and altruism (β = 0.207, *p* < 0.01) were the two most important motivations, both of which were positively associated with young adults’ content creation intention in ADOCs. Second, perceived enjoyment (β = −0.08, *p* < 0.05) was negatively associated with young adults’ content creation intention in ADOCs. Reward motivation (β = −0.032, *p* > 0.05) was not significantly associated with young adults’ content creation in ADOCs. Therefore, hypothesis H9 was not supported. 

#### 5.2.2. Moderating Effect Test

We added empathy as a moderating variable to the original model to study the moderating effect of empathy on the relationships among anxiety venting, perceived enjoyment, online communities’ sense of belonging, altruism, self-efficacy, anxiety information acquisition, reciprocity motivation, reward motivation, social motivation, and young adults’ content creation intention in ADOCs. Empathy was interacted with the latent variables in the model to test the effects of the interaction items on young adults’ content creation intention in ADOCs. The test results are shown in Table 6. Empathy (β = −0.128, *p* < 0.01) negatively moderated the relationships between self-efficacy and young adults’ content creation intention in ADOCs, suggesting that hypothesis H11-5 was supported. In addition, it can be seen from Table 6 that the interaction items in hypotheses H11-1, H11-2, H11-3, H11-4, H11-6, H11-7, H11-8, and H11-9 were not significantly associated with young adults’ content creation intention in ADOCs, and these hypotheses were not supported. 

## 6. Conclusions and Contributions

Based on self-determination theory, this paper uses the partial least square (PLS) structural equation model to empirically test young adults’ content creation behavioural motivation in ADOCs. The results lead to the following conclusions:

(1) In terms of intrinsic motivation, anxiety venting, perceived enjoyment, online communities’ sense of belonging, altruism, and self-efficacy are all significantly related to young adults’ content creation intention in ADOCs. Among these motivations, anxiety venting is the most significant motivation, indicating that, for young adults, the main motivation underlying their content creation behaviour in ADOCs is anxiety venting. Proper venting can alleviate the nervousness, fear, and anxiety caused by anxiety disorders to some extent and can also improve the activity of the community. Only perceived enjoyment motivation is negatively associated with young adults’ content creation intention in ADOCs. Generally, users participate in online communities for fun and pleasure. Therefore, this motivation usually has positive effects. However, considering the particularities of young adults with anxiety disorders, the authors determined that this effect occurs because young adults in ADOCs can alleviate their anxiety by posting comments and engaging in other content creation behaviours; therefore, young adults in ADOCs are more likely to display the intention to create content, such as posting comments, when the perceived enjoyment is low.
(2) In terms of extrinsic motivation, anxiety information acquisition, reciprocal motivation, and social motivation are positively related to young adults’ content creation intention in ADOCs. Among these motivations, anxiety information acquisition and reciprocal motivation are the most significant, which means that the main external factor that promotes young adults’ content creation behaviour in ADOCs is obtaining information related to the anxiety disorders. According to the actual experience of participating in ADOCs, it is found that many posts in ADOCs focus on the medication status of patients and their illness and treatment process; furthermore, these posts also have more comments and replies. Therefore, combined with the research, it is found that the managers of ADOCs, such as the hosts of the microblog anxiety disorder chat and the owners of Baidu anxiety disorder Tieba, can promote community activity and communication among users by sharing mental health information related to anxiety disorders. However, reward motivation is not significantly related to young adults’ content creation intention in ADOCs, indicating that, for young adults, rewards such as money or community points do not motivate them to create content such as posts, comments, and so on.
(3) Regarding the moderating effect, empathy only negatively moderates the relationships between self-efficacy and young adults’ content creation intention in ADOCs, while the other interaction items have no significant moderating effect. The authors determined that self-efficacy reflects one’s self-confidence and tendency towards rational thinking, while empathy reflects one’s emotional empathy for others and tendency towards perceptual thinking. Therefore, among those with high empathy, emphasising self-efficacy will reduce young adults’ content creation intention. In terms of the mediating effect, young adults’ content creation intention in ADOCs is positively related to their content creation behaviour, indicating that content creation behaviour is more likely to occur when young adults have a high content creation intention.

This study makes several significant contributions to the current scholarly literature. First, this study not only discusses the motivation underlying young adults’ content creation behaviour in ADOCs but also analyses its regulating effect on the content creation behavioural motivation of young adults from the perspective of empathy, which broadens research in the fields of empathy and behavioural science and provides a new research direction. In addition, this study combines self-determination theory and the theory of planned behaviour to analyse the internal and external motivations underlying young adults’ content creation behaviours. Self-determination theory is widely used to study employees’ work motivation [66,67], students’ learning motivation [68], etc. However, it is rarely adopted when studying the behaviour of users in anxiety disorder online communities. This research applies self-determination theory to study the behavioural motivations of young adults in ADOCs, which enriches the application scope of the theory. Therefore, this research has some theoretical implications. 

This study also has some practical significance for the construction and management of anxiety disorder online mental health communities and other communities of the same nature. First, studying the behavioural motivations of young adults’ content creation in ADOCs can lead to a better understanding of the reasons that they post, reply, consult, and forward within such a community. Based on the research results, ADOCs can be improved in a targeted manner. For example, based on anxiety information acquisition motivation, the managers of ADOCs, such as the hosts of the microblog anxiety disorder chat and the owners of Baidu anxiety disorder Tieba, can regularly share mental health information related to anxiety disorders. In addition, ADOCs can set up an emotional expression platform, where users can vent their negative emotions and receive encouragement and comfort from others. These measures can increase the activity of community users and promote the success and sustainable development of the community. Regarding the sense of belonging and social motivation, ADOCs can set up a friendship section for individuals who want to connect with others in the community. This could provide them with a more convenient channel to form social relationships. Furthermore, the results can also promote communication among groups of young adults with anxiety disorders and help them to find ways to relieve and improve their symptoms during the communication process, which has certain practical significance. Although this research has certain theoretical and practical significance, it also has certain limitations. For example, this article is limited to consideration of only online health communities for anxiety disorders. Future research can also consider online health communities focused on multiple mental illnesses. 

## Figures and Tables

**Table 1:** Questionnaire variables, items, and sources.

Variables	Name	Items	Sources
Anxiety Venting	AV1	Posting helps me get rid of the negative emotions caused by my illness	[54]
	AV2	I am happy to vent the negative emotions caused by anxiety	
Perceived Enjoyment	PE1	I feel relaxed	[31]
	PE2	The process of participating in the topic is enjoyable	
	PE3	I enjoy being involved	
Online Communities’ Sense of Belonging	OCSB1	I feel strongly that I am a member of the community	[55]
	OCSB2	I think I have a strong psychological and emotional connection with this community	
	OCSB3	I am happy to be a member of the community	
	OCSB4	I want to contribute to the atmosphere and activity of the community	
Altruism	AL1	I would like to help others in the community	[17,36]
	AL2	I am happy to help others of the community	
	AL3	I enjoy helping others solve problems, which gives me a sense of accomplishment	
	AL4	Helping other community users makes me happy	
Self-Efficacy	SE1	I believe I can provide other users with useful content and knowledge	[56]
	SE2	I have the ability, experience and advice to solve problems for other users	
	SE3	I am confident to comment on and respond to users’ posts	
Anxiety Information Acquisition	AIA1	I consult on the relief and treatment of anxiety	[57]
	AIA2	Discussing with other users will help me make decisions	
	AIA3	Browsing posts and interactions give me some inspiration	
	AIA4	I often participate in the community to get information about anxiety disorders	
Reciprocity Motivation	RE1	I believe I will get a response when posting, commenting, replying, and consulting	[38]
	RE2	I hope someone can respond when I need it	
	RE3	When sharing knowledge and experience, I hope to get knowledge and advice when needed	
	RE4	When I consult, I believe my question will be answered in the future	
Reward Motivation	RM1	I expect money in return	[58]
	RM2	I want to increase my personal points	
Social Motivation	SM1	I can make friends with members of the community	[59]
	SM2	I can meet people with similar experiences and psychological states	
	SM3	I can get support and encouragement from other members	
	SM4	I can communicate with people with similar ideas	
Empathy	EM1	I try my best to see the world through the eyes of community members	[60]
	EM2	I can imagine the feelings of community members	
	EM3	I try to understand the psychology of community members	
	EM4	I try to see the problem from the perspective of others	
Young Adults’ Content Creation Intention	YACCI1	I am willing to post, comment, consult, forward, etc.	[61]
	YACCI2	When other users in the community @ or comment on me, I intend to respond	
	YACCI3	I will comment or post in the community for consultation, etc.	
Young Adults’ Content Creation Behaviour	YACCB1	I often post content in the community (such as consulting, commenting, posting and forwarding).	[62]
	YACCB2	I usually spend a lot of time posting content in the community	
	YACCB3	When participating in the community, I actively post content	
	YACCB4	I often engage in discussions on various anxiety topics, rather than specific topics	

**Table 2:** Sample demographic data.

Demographic Characteristics		Frequency	Percentage
Gender	Male	180	53.8%
	Female	154	46.2%
Education	High school degree and below	139	41.6%
	Associate degree	16	4.8%
	Bachelor degree	117	35%
	Master degree and above	62	18.6%
Living area	First-tier cities	36	10.8%
	Second-tier cities	36	10.8%
	Third-tier cities	72	21.6%
	Fourth-tier cities and below	190	56.8%

**Table 3:** Results of reliability and aggregate validity.

		Reliability		Convergent Validity	
Variable	Factor	Factor Loading	Cronbach’s Alpha	CR	AVE
AL	AL1	0.919	0.917	0.942	0.802
	AL2	0.909			
	AL3	0.856			
	AL4	0.897			
YACCB	YACCB1	0.93	0.916	0.94	0.796
	YACCB2	0.835			
	YACCB3	0.917			
	YACCB4	0.884			
YACCI	YACCI1	0.942	0.899	0.937	0.833
	YACCI2	0.883			
	YACCI3	0.912			
EM	EM1	0.954	0.936	0.954	0.84
	EM2	0.895			
	EM3	0.912			
	EM4	0.904			
AIA	AIA1	0.869	0.875	0.915	0.729
	AIA2	0.866			
	AIA3	0.878			
	AIA4	0.799			
PE	PE1	0.944	0.922	0.951	0.866
	PE2	0.93			
	PE3	0.917			
RE	RE1	0.845	0.883	0.919	0.739
	RE2	0.879			
	RE3	0.878			
	RE4	0.837			
RM	RM1	0.979	0.957	0.979	0.959
	RM2	0.98			
OCSB	OCSB1	0.839	0.835	0.889	0.668
	OCSB2	0.841			
	OCSB3	0.729			
	OCSB4	0.855			
SE	SE1	0.948	0.937	0.96	0.888
	SE2	0.937			
	SE3	0.942			
SM	SM1	0.859	0.908	0.935	0.783
	SM2	0.88			
	SM3	0.897			
	SM4	0.904			
AV	AV1	0.9	0.726	0.879	0.785
	AV2	0.871			

**Table 4:** Correlation results of the scale.

	Discriminant Validity											
	AL	YACCB	YACCI	EM	AIA	PE	RE	RM	OCSB	SE	SM	AV
AL	**0.895**											
YACCB	0.193	**0.892**										
YACCI	0.739	0.266	**0.913**									
EM	0.188	0.189	0.27	**0.916**								
AIA	0.645	0.205	0.703	0.3	**0.854**							
PE	0.232	0.261	0.239	0.266	0.262	**0.93**						
RE	0.641	0.216	0.672	0.337	0.612	0.278	**0.86**					
RM	0.107	0.36	0.16	0.328	0.144	0.513	0.183	**0.979**				
OCSB	0.672	0.231	0.68	0.234	0.605	0.331	0.591	0.273	**0.817**			
SE	0.532	0.171	0.607	0.17	0.552	0.283	0.508	0.292	0.567	**0.942**		
SM	0.548	0.208	0.614	0.342	0.606	0.315	0.61	0.245	0.537	0.516	**0.885**	
AV	0.605	0.226	0.676	0.258	0.566	0.386	0.5	0.26	0.561	0.477	0.468	**0.886**

Note: The diagonal bold values represent the square root of AVE, and the lower triangle is the Pearson correlation coefficient.

**Table 5:** Main effect test results.

Hypothesis	Path	β	T-Statistic	Supported?
H2	AV ≥ YACCI	0.249 **	5.659	Supported
H3	PE ≥ YACCI	−0.077 *	2.077	Supported
H4	OCSB ≥ YACCI	0.131 *	2.39	Supported
H5	AL ≥ YACCI	0.207 **	3.357	Supported
H6	SE ≥ YACCI	0.127 *	2.563	Supported
H7	AIA ≥ YACCI	0.151 **	2.572	Supported
H8	RE ≥ YACCI	0.159 **	3.245	Supported
H9	RM ≥ YACCI	−0.032	0.746	Not supported
H10	SM ≥ YACCI	0.092 *	2.126	Supported
H1	YACCI ≥ YACCB	0.525 **	5.659	Supported

Notes: * *p* < 0.05; ** *p* < 0.01.

**Table 6:** Results of regulatory effect.

Hypothesis	Path	β	T-Statistic	Supported?
H11-1	AV × EM ≥ YACC1	−0.026	0.589	Not supported
H11-2	PE × EM ≥ YACCI	0.009	0.226	Not supported
H11-3	OCSB × EM ≥ YACCI	0.081	1.469	Not supported
H11-4	AL × EM ≥ YACCI	0.053	0.882	Not supported
H11-5	SE × EM ≥ YACCI	−0.128 **	2.758	Supported
H11-6	AIA × EM ≥ YACCI	0.044	0.618	Not supported
H11-7	RE × EM ≥ YACCI	−0.055	1.035	Not supported
H11-8	RM × EM ≥ YACCI	0.008	0.202	Not supported
H11-9	SM × EM ≥ YACCI	−0.02	0.441	Not supported

Note: ** *p* < 0.01.

## Data Availability

No additional data are available.

## Abbreviations

The following abbreviations are used in this manuscript:

ADOCs	Anxiety disorder online communities
WHO	World Health Organization
OHCs	Online health communities
SDT	Self-determination theory
PLS	Partial least squares
AVE	Average variance extracted
CR	Composite reliability
AV	Anxiety venting
PE	Perceived enjoyment
OCSB	Online communities’ sense of belonging
AL	Altruism
SE	Self-efficacy
AIA	Anxiety information acquisition
RE	Reciprocity motivation
RM	Reward motivation
SM	Social motivation
EM	Empathy
YACCI	Young adults’ content creation intention
YACCB	Young adults’ content creation behaviour
